# Early Impairment of Paracrine and Phenotypic Features in Resident Cardiac Mesenchymal Stromal Cells after Thoracic Radiotherapy

**DOI:** 10.3390/ijms25052873

**Published:** 2024-03-01

**Authors:** Vittorio Picchio, Roberto Gaetani, Francesca Pagano, Yuriy Derevyanchuk, Olivia Pagliarosi, Erica Floris, Claudia Cozzolino, Giacomo Bernava, Antonella Bordin, Filipe Rocha, Ana Rita Simões Pereira, Augusto Ministro, Ana Teresa Pinto, Elena De Falco, Gianpaolo Serino, Diana Massai, Radia Tamarat, Maurizio Pesce, Susana Constantino Rosa Santos, Elisa Messina, Isotta Chimenti

**Affiliations:** 1 Department of Angio Cardio Neurology, IRCCS Neuromed, 86077 Pozzilli, Italy; vittorio.picchio@uniroma1.it; 2 Department of Molecular Medicine, Sapienza University, 00161 Roma, Italy; roberto.gaetani@uniroma1.it (R.G.); derevyanchuk.1679469@studenti.uniroma1.it (Y.D.); pagliarosi.1596107@studenti.uniroma1.it (O.P.); 3 Institute of Biochemistry and Cell Biology, National Council of Research (IBBC-CNR), 00015 Monterotondo, Italy; francesca.pagano@cnr.it; 4 Department of Medical Surgical Sciences and Biotechnologies, Sapienza University, 04100 Latina, Italy; erica.floris@uniroma1.it (E.F.); claudia.cozzolino@uniroma1.it (C.C.); antonella.bordin@uniroma1.it (A.B.); elena.defalco@uniroma1.it (E.D.F.); 5 Centro Cardiologico Monzino, IRCCS, 20138 Milano, Italy; bernavag@yahoo.com (G.B.); maurizio.pesce@cardiologicomonzino.it (M.P.); 6 Centro Cardiovascular da Universidade de Lisboa (CCUL@RISE), Lisbon School of Medicine, Universidade de Lisboa, 1649-028 Lisbon, Portugal; filipenunesrocha@gmail.com (F.R.); ritasimoespereira@gmail.com (A.R.S.P.); augusto.ministro@gmail.com (A.M.); anapinto@ua.pt (A.T.P.); sconstantino@medicina.ulisboa.pt (S.C.R.S.); 7 Mediterranea Cardiocentro, 80122 Napoli, Italy; 8 Department of Mechanical and Aerospace Engineering, Politecnico di Torino, 10129 Torino, Italy; gianpaolo.serino@polito.it (G.S.); diana.massai@polito.it (D.M.); 9 Interuniversity Center for the Promotion of the 3Rs Principles in Teaching and Research, 10129 Torino, Italy; 10 Institut de Radioprotection et de Sûreté Nucléaire (IRSN), 92260 Fontenay-aux-Roses, France; radia.tamarat@irsn.fr

**Keywords:** cardiotoxicity, radiotherapy, radiation-induced cardiomyopathy, radiation-induced heart disease, cardiac fibrosis, cardiac stromal cells

## Abstract

Radiotherapy-induced cardiac toxicity and consequent diseases still represent potential severe late complications for many cancer survivors who undergo therapeutic thoracic irradiation. We aimed to assess the phenotypic and paracrine features of resident cardiac mesenchymal stromal cells (CMSCs) at early follow-up after the end of thoracic irradiation of the heart as an early sign and/or mechanism of cardiac toxicity anticipating late organ dysfunction. Resident CMSCs were isolated from a rat model of fractionated thoracic irradiation with accurate and clinically relevant heart dosimetry that developed delayed dose-dependent cardiac dysfunction after 1 year. Cells were isolated 6 and 12 weeks after the end of radiotherapy and fully characterized at the transcriptional, paracrine, and functional levels. CMSCs displayed several altered features in a dose- and time-dependent trend, with the most impaired characteristics observed in those exposed in situ to the highest radiation dose with time. In particular, altered features included impaired cell migration and 3D growth and a and significant association of transcriptomic data with GO terms related to altered cytokine and growth factor signaling. Indeed, the altered paracrine profile of CMSCs derived from the group at the highest dose at the 12-week follow-up gave significantly reduced angiogenic support to endothelial cells and polarized macrophages toward a pro-inflammatory profile. Data collected in a clinically relevant rat model of heart irradiation simulating thoracic radiotherapy suggest that early paracrine and transcriptional alterations of the cardiac stroma may represent a dose- and time-dependent biological substrate for the delayed cardiac dysfunction phenotype observed in vivo.

## 1. Introduction

Radiotherapy (RT) represents a main form of cancer treatment at almost every stage. In some types of cancer, such as breast cancer, most patients are irradiated as part of their therapy (source: cancerresearchuk.org). It is well known that RT may cause an increased risk for clinically relevant late side effects in surrounding organs/tissues, impacting the quality of life and survival of patients [1,2]. Thoracic RT often leads to coincidental irradiation of the heart, increasing the risk of developing a wide range of radiation-induced heart diseases (RIHDs), including ischemic heart disease, pericardial and myocardial fibrosis, and congestive heart failure. Thus, minimizing therapeutic morbidity has become a major challenge of concern. Technological improvements have allowed a significant reduction of RT doses affecting cardiac tissue. However, there seems to be no minimal safe dose to avoid cardiac complications [3,4,5,6,7,8], and RIHDs remain potential severe late complications for cancer survivors [9].

The progressive mechanisms involved in cardiotoxicity induced by therapeutic radiation doses are still largely unknown. Radiation damage to tissues is primarily due to the generation of free radicals that damage DNA, hinder replication and protein synthesis, and enhance pro-inflammatory signals [10]. Moreover, radiation-induced vascular injury and perivascular fibrosis lead to inflammatory and thrombotic events, focal ischemia, and interstitial fibrosis [11,12]. Cardiomyocytes, as perennial cells accounting for the majority of the heart [13], are relatively resistant to radiation damage. In contrast, non-cardiomyocytes, including cardiac mesenchymal stromal cells (CMSCs), represent the majority of proliferating cells that may be susceptible to radiation. In fact, chronic low RT doses can potentially expose the stromal compartment (e.g., fibroblasts, mesenchymal cells, pericytes, etc.) to low-grade insults and phenotypic changes. CMSCs include multiple cell types and functional states. They play numerous key roles in homeostasis and disease, such as regulating extracellular matrix (ECM) deposition and angiogenesis [14,15]. CMSCs also provide paracrine signaling involved in the stress response, crosstalk with immune cells [16], and cardioprotective mechanisms [17,18]. In contrast, the microenvironment can condition the CMSC phenotype. Several stimuli or insults have been described to induce a phenotypic shift in CMSCs toward profibrotic (e.g., ECM production, stiffness enhancement) and/or proinflammatory functions (e.g., cytokine/chemokine release), including ischemia, chronic ECM remodeling, and metabolic stress [19,20,21,22]. Therefore, compared to cardiomyocytes, with their relatively long lag in manifesting dysfunction due to RT, stromal cells may represent an early target and/or mediator of radiation-induced cardiac damage. Indeed, early analysis of RT-specific effects on resident CMSCs is still missing.

Recently, an innovative rat model of RT-induced cardiotoxicity has been described [23]. This clinically accurate and relevant model has uncovered unprecedented insights into the mid- to long-term occurrence of cardiac dysfunction due to RT. Rats were irradiated with fractionated doses to the heart (cumulative doses of 0.92, 6.9, or 27.6 Gy, with appropriate controls) and followed up to 18 months after the end of RT. Molecular cardiac alterations are detectable 7 months post-RT at the highest doses, such as altered protein levels of cTnT, Myosin6, and Calsequestrin2, and altered gene expression of *Tgfb2* and *Sod2*. A dose-dependent diastolic dysfunction, in terms of reduced global longitudinal strain (GLS), is developed at 12 months post-RT, and this trend becomes directly proportional to the RT dose 18 months after the end of RT. This model appears highly relevant from a translational perspective. First, it perfectly resembles the long pathogenetic lag observed in cancer patients, where cardiac diseases usually arise over 10 years after RT, although it may appear at lower threshold values in elderly patients [24]. Importantly, GLS is impaired in patients with heart failure with preserved ejection fraction (HFpEF) and is considered suggestive of subclinical cardiotoxicity [25].

The aim of the present study was to investigate the effects of different RT cardiac doses on the phenotypic and functional features of resident CMSCs, exploiting the clinically relevant rat model of fractionated heart irradiation with delayed cardiac dysfunction. Stromal cells are the most abundant replicating cardiac cell type with high angiogenic, trophic, and immune-modulating paracrine activity [18], most likely sensitive to low-dose radiation. We hypothesized that the RT-induced phenotypic and paracrine changes in CMSCs may be an early sign and/or mechanism of RT-induced cardiac toxicity involved in microenvironmental signaling, affecting long-term cardiac fibrosis, hypertrophy, and diastolic stiffness, leading to HFpEF [24].

## 2. Results

**Figure 1:**
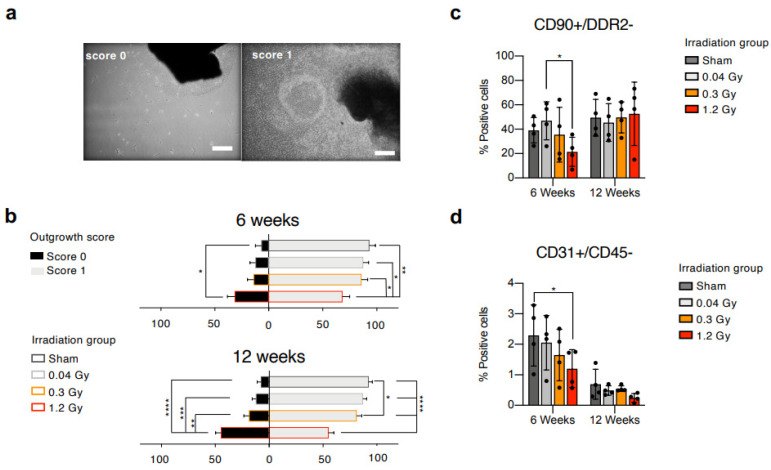
Characterization of explant outgrowth and flow cytometry. (**a**) Representative microscopy images of primary culture for the different explant outgrowth scores. Score 0 = no or few cells within a 200 nm radius; score 1 = confluent cell outgrowth beyond a 200 nm radius. Scale bar = 500 µm. Average outgrowth score analysis of primary explant cultures (at least 30 fields per explant) is reported from rat hearts at 6-week and 12-week follow-up (**b**). Quantification by flow cytometry was performed on the CD90+/DDR2− mesenchymal/stromal nonactivated subpopulation (**c**) and the CD31+/CD45− endothelial-like fraction (**d**) at the 6- and 12-week follow-ups. N >= 4 per condition. * = *p* < 0.05, ** = *p* < 0.01, *** = *p* < 0.001, **** = *p* < 0.0001. ANOVA was used for statistical analysis.

Rats were exposed to fractionated heart irradiation at different clinically relevant doses, with 23 daily fractions of 0.04 Gy, 0.3 Gy, or 1.2 Gy, as previously described [23]. Animals from the sham group underwent all related procedures, receiving a 0.0 Gy dose. Hearts were explanted 6 or 12 weeks after the end of irradiation, and myocardial tissue was plated as explant cultures. Primary explant cultures from all experimental groups were visually analyzed 3 weeks after plating and evaluated by a blinded operator with the outgrowth score system described in the methods section. Representative images of the scores are presented in Figure 1a. Cell cultures derived from rats 6 weeks after the end of RT (Figure 1b) displayed a trend toward a lower proportion of explants with score 1 (confluent cell outgrowth beyond 200 µm radius) at higher RT doses, with a significantly reduced percentage for the 1.2 Gy versus all groups. Cell cultures derived from animals at the 12-week follow-up (Figure 1b), instead, showed a significantly higher proportion of score 0 explants (no or few cells within a 200 µm radius) and a significantly lower percentage of score 1 explants in the 1.2 Gy group compared to all other groups. This early observation suggested a combination of reduced proliferation and migration capabilities of the explant-derived cells from cardiac tissue subjected to the highest RT dose at both follow-up times.

Explant-derived cells were collected and analyzed by flow cytometry. We applied a staining panel including hematopoietic (CD45), mesenchymal/stromal (CD90), activated fibroblasts or myofibroblasts (DDR2), and endothelial (CD31) markers, and designed gating strategies to analyze subpopulations with different phenotypes. All samples had a negligible proportion of CD45+ cells (<5%), confirming their non-hematopoietic nature (Supplemental Figure S1a,b). All further analyses were performed on the CD45-negative (CD45−) fraction.

The results of the 6-week follow-up (Figure 1c, Supplemental Table S1) show that CMSCs isolated from the high-dose group (1.2 Gy) contained a significantly reduced proportion of CD90+/DDR2− mesenchymal non-activated cells compared to the low-dose group (0.04 Gy). Similarly, the CD45−/CD31+ endothelial-like subpopulation (Figure 1d), important for its role in response to injury [13], displayed a clear trend toward reduction at increasing RT doses, reaching statistical significance between sham and 1.2 Gy. The results of the 12-week follow-up show that the percentage of CD90+/DDR2− CMSCs in the 1.2 Gy group returned to similar percentages compared to the other groups (Figure 1c). A reduction with time in the CD45−/CD31+ subset was detectable in all groups, but the trend of decreasing percentages at increasing RT doses persisted (Figure 1d, Supplemental Table S1). Overall, the data suggest an early reduction in the pool of non-activated CMSCs associated with higher RT exposure.

**Figure 2:**
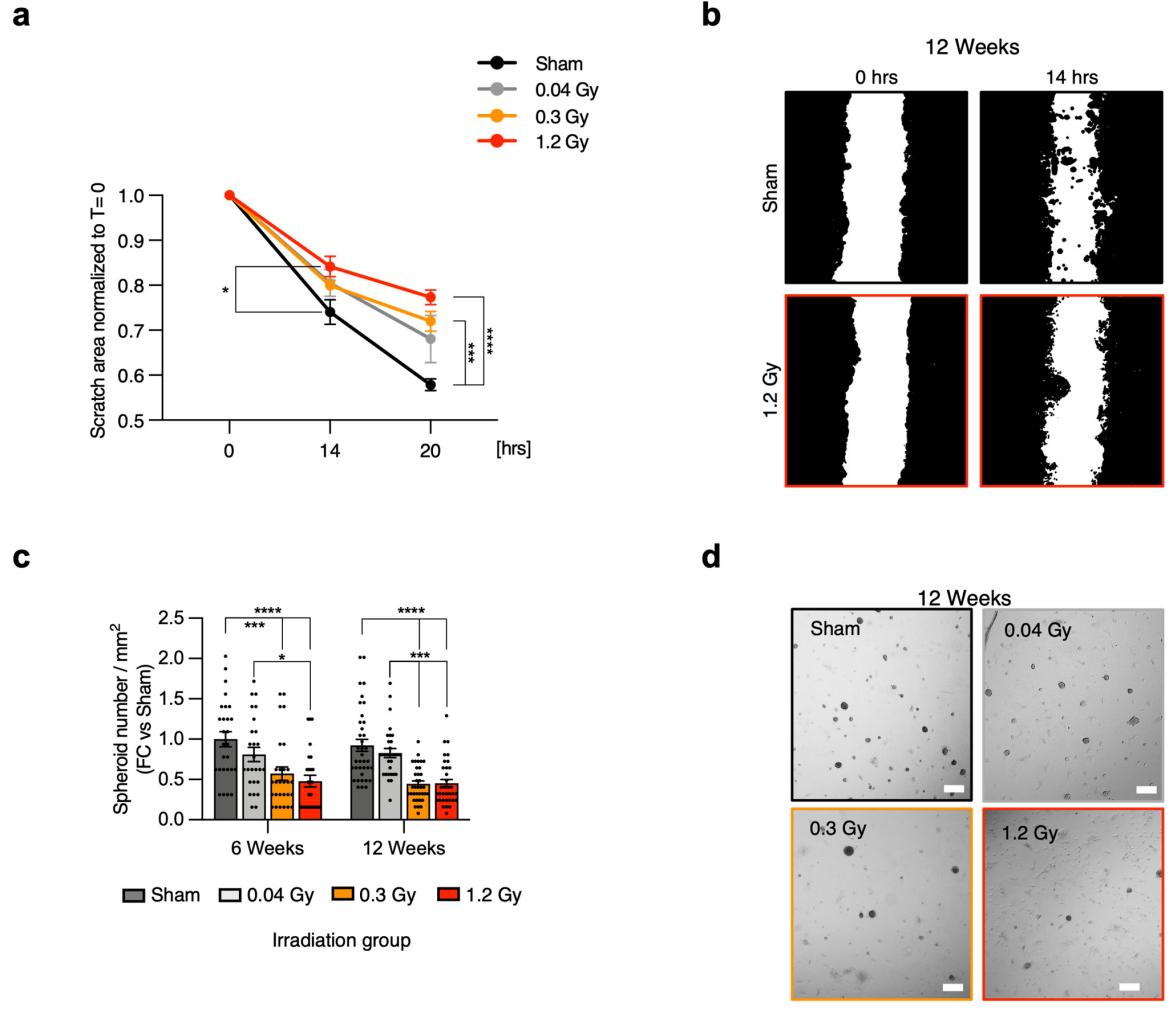
Cell migration and spheroid growth capacity of cardiac stromal cells. Relative quantification of the migration assay at 0, 14, and 20 h post-scratch for CMSCs isolated from all dose groups (**a**), with representative analysis masks of sham and 1.2 Gy images after 14 h (**b**). The migration data were normalized to the scratch area at time 0 h. N (rats for cell derivation) = 4. (**c**) Quantification of the spheroid-forming assay performed on CMSCs from both follow-up times and representative microscopy images of the 12-week follow-up (**d**). Scale bar = 100 µm. N (analyzed spheroids) >30 for each group. * = *p* < 0.05, *** = *p* < 0.001, **** = *p* < 0.0001. ANOVA was used for statistical analysis.

Next, CMSC proliferation was evaluated by a metabolic activity assay. The results from both the 6- and 12-week follow-up points did not show any detectable difference among groups (Supplemental Figure S2A,B). Migration was then specifically assessed by scratch assay, as it represents a key functional feature of mesenchymal cells. Cells with the most altered explant outgrowth profile were analyzed, that is, CMSCs from the 12-week follow-up point. The results confirmed a progressive impairment at increasing RT doses (Figure 2a,b, Supplemental Figure S3). In detail, a significant reduction in the migration capacity of CMSCs from the 1.2 Gy group versus sham was detected after 14 h. Moreover, CMSCs from both the 0.3 Gy and 1.2 Gy groups displayed significantly reduced migration capacity versus sham after 20 h. These results were consistent with the outgrowth score assessment and suggested that increasing RT doses may cause early impairment of the migration capacity of CMSCs.

We further characterized CMSCs for another mesenchymal feature, which is the capacity for spontaneous 3D growth, which is considered in the literature as a functional feature of undifferentiated and mesenchymal cells [26,27]. At the 6-week follow-up point (Figure 2c), the results showed a significant decrease in the spheroid-forming ability of CMSCs from the 1.2 Gy group versus sham and 0.04 Gy and from the 0.3 Gy group versus sham. Interestingly, at the 12-week follow-up (Figure 2c,d), CMSCs from both the 0.3 Gy and 1.2 Gy groups displayed significantly reduced spheroid-forming capacity versus both sham and 0.04 Gy, suggesting a decline in this typical mesenchymal functional feature. There were no detectable differences in spheroid size among groups (Supplemental Figure S2C,D), which was consistent with the proliferation assay.

**Figure 3:**
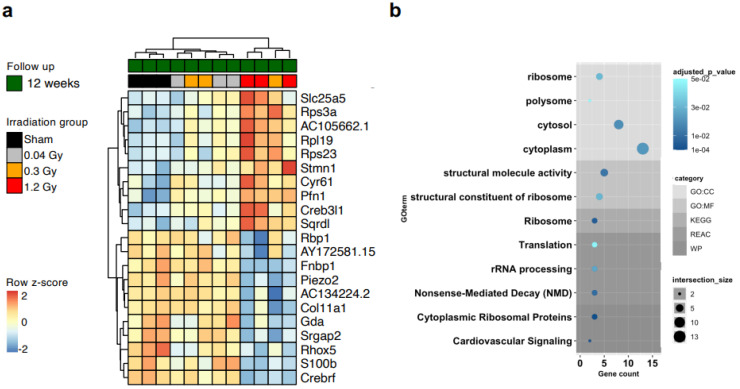
Transcriptomic dose-dependent analysis of CMSCs. Heatmap with hierarchical clustering (**a**) of the significantly modulated genes between the cells of the sham and 1.2 Gy groups at the 12-week follow-up, with the relative gene ontology (GO) analysis output (**b**), evidencing GO categories, *p* values, and gene counts per category. Only genes with significant adjusted *p* values were selected.

**Figure 4:**
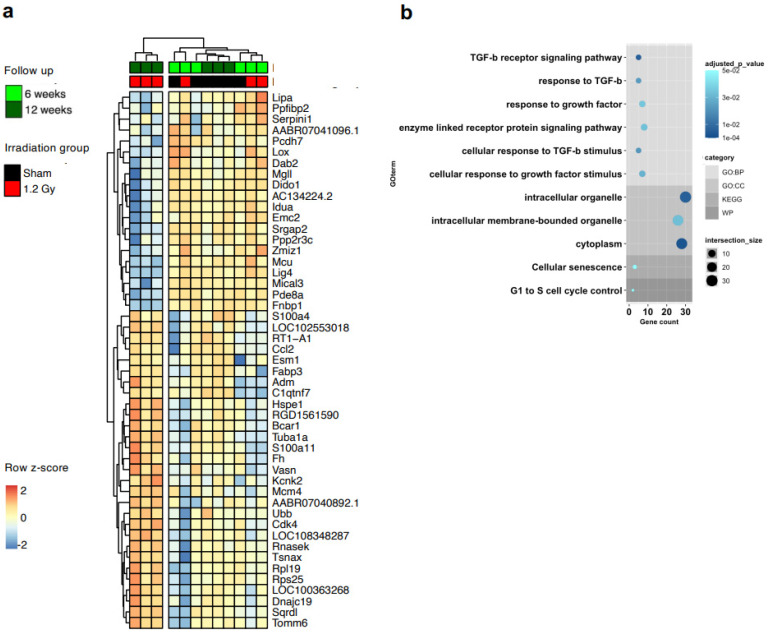
Transcriptomic time-dependent analysis of CMSCs. Heatmap with hierarchical clustering (**a**) of the genes significantly modulated with time of follow-up in cells from the 1.2 Gy group, with the relative gene ontology (GO) analysis output (**b**), evidencing GO categories, *p* values, and gene counts per category. Only genes with significant adjusted *p* values were selected.

We screened by transcriptomic analysis samples of whole heart tissue, but we could not detect any global differential profile (Supplemental Figure S4), consistent with the absence of any early phenotype in the rat model [23]. Thus, we performed transcriptomic analysis directly on CMSCs to unravel cell-type-specific changes. RNAseq analysis revealed 21 genes that were significantly modulated between the sham and 1.2 Gy groups at the 12-week follow-up (adjusted *p* < 0.05), with clear clustering of the cells from the high-dose group (Figure 3a). The corresponding gene ontology (GO) analysis returned quite generic terms, mostly related to cellular components (Figure 3b, Supplemental Table S2). The differential expression analysis returned a second group of 49 genes that were significantly modulated (adjusted *p <* 0.05) in the 1.2 Gy group according to the time of follow-up, between 6 and 12 weeks (Figure 4a). We validated the RNA-seq data by RT-PCR (Supplemental Figure S5). Interestingly, the GO analysis of this set returned several biological process terms with significant adjusted p values, including highly significant terms related to the key fibrotic pathway of TGFβ1 signaling (Figure 4b, Supplemental Table S3).

With this premise, we analyzed markers and pathways of myofibroblast differentiation and function. Soluble collagen release was evaluated without detecting any modulation between groups (Supplemental Figure S6a). Since some of the myofibroblast features are known to be stiffness-dependent [28], we plated the cells on a polydimethylsiloxane (PDMS) micropillar substrate with a stiffness of 20, 100 (roughly corresponding to the fibrotic myocardium), or 1000 kPa. The percentage of alpha-smooth muscle actin (αSMA)-positive cells was assessed by immunofluorescence as a hallmark of myofibroblast differentiation, showing a trend of increasing percentages of positive cells in CMSCs at increasing RT doses on 100 kPa stiffness (Supplemental Figure S6b). We also verified the differential ability of the cells to translate mechanically activated signals into Yes-Associated Protein 1 (YAP)-dependent gene expression as another myofibroblast feature. No significant differences could be detected in the cytoplasm-to-nucleus ratio of YAP, although a trend of increasing ratio at decreasing stiffness could be observed in CMSCs at the highest RT dose (1.2 Gy) (Supplemental Figure S6d). The gene expression modulation trends of the YAP target genes *ACTA2*, *ANKDR1*, *CTGF*, and *CYR61* at different stiffness values were comparable among all groups (Supplemental Figure S6e), consistent with the similar YAP nuclear localization. Overall, the results suggest that the altered features observed in CMSCs 12 weeks after RT are not yet associated with altered phenotypic features of myofibroblast differentiation in our conditions.

**Figure 5:**
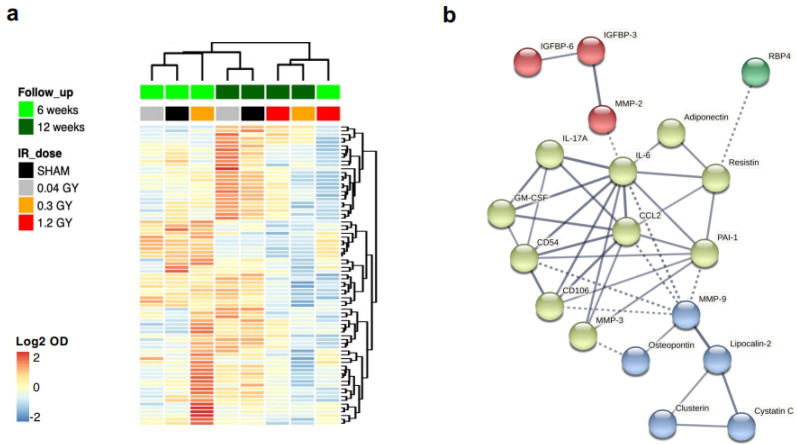
Analysis of the differential release of cytokines. Heatmap with hierarchical clustering of Euclidean distance (**a**), calculated on Log2 values from normalized optical density (OD) of protein arrays on conditioned media pool. (**b**) Association network obtained on the STRING database for the shortlist of 30 cytokines (top 15 upregulated and top 15 downregulated). Only associations with a strength >0.7 are shown. Colors define different clusters identified by the k-means method; dotted lines connect cytokines in different clusters.

It is well known that CMSCs regulate myocardial physiopathology through paracrine functions [18]. Interestingly, significant GO terms for biological processes related to growth factor and cytokine responses also emerged from the bioinformatic analysis (Figure 4b, Supplemental Table S3). Thus, we analyzed in more detail the differential paracrine profile of CMSCs by protein arrays on conditioned media of cell cultures at each follow-up and radiation dose and plotted the log2 normalized optical density values (Figure 5a). Hierarchical clustering shows that the paracrine profiles of cells from the sham, 0.04 Gy, and 0.3 Gy dose groups at the 6-week follow-up clustered together and were globally similar to the sham and 0.04 Gy groups at the 12-week follow-up. The 1.2 Gy groups from both follow-up times and the 0.3 Gy group at the 12-week follow-up clustered together instead and had an overall reduction in most of the secreted cytokines detected.

A shortlist of the 30 most modulated cytokines in the 1.2 Gy dose group with time (6 weeks versus 12 weeks, top 15 upregulated + top 15 downregulated) was loaded on the STRING database to create the functional association network (Figure 5b). Multiple GO terms were obtained with high strength (>1, i.e., over 10-fold enrichment) and a significant false discovery rate (FDR < 0.05) (Supplemental Table S4). The association of cytokines with a selection of GO terms of interest (e.g., linked to migration, inflammation, or response to radiation) is shown superimposed on the STRING network graph in Supplemental Figure S7. Overall, the paracrine profiles indicated reduced cytokine release by CMSCs with increasing RT doses and longer follow-up times.

**Figure 6:**
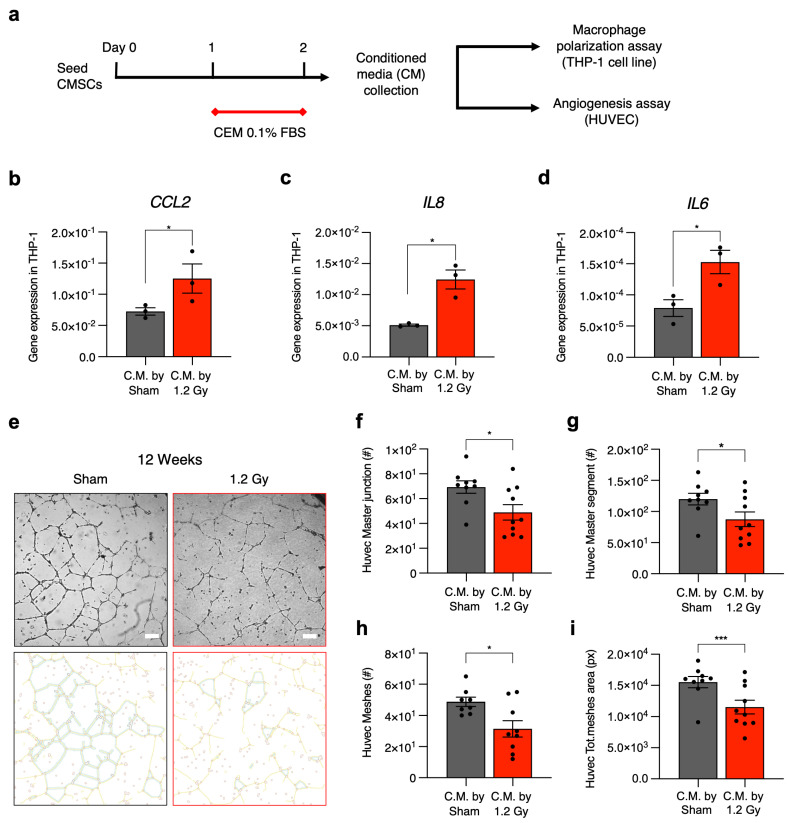
Analysis of functional paracrine effects of conditioned media. Experimental design (**a**). M1 polarization of THP1 monocyte cells cultured with conditioned media (C.M.) of CMSCs from the 12-week follow-up was evaluated by real-time qPCR analysis for the markers *CCL2* (**b**), *IL6* (**c**), and *IL8* (**d**). N = 3. Representative images of the tube-forming assay with human umbilical vein endothelial cells (HUVECs) exposed to CMSC-C.M. are reported (**e**), together with the quantification of master junctions (**f**), master segments (**g**), meshes (**h**), and total mesh area (**i**). Scale bar = 200 µm. * = *p* < 0.05, *** = *p* < 0.001. A *t* test was used for statistical analysis.

The differences in CMSC secretomic profiles were validated with functional paracrine assays on two fundamental cell types affected by stromal cells in situ in the myocardium, namely macrophages and endothelial cells [15] (Figure 6a). We used media conditioned by CMSCs of the 1.2 Gy group at the 12-week follow-up, which displayed the most significant modulation of the paracrine profile. Medium conditioned by CMSCs of the sham group at the 12-week follow-up was used as a control. Gene expression analysis in the treated THP-1 macrophage cell line showed that exposure to media conditioned by CMSCs from irradiated rats significantly increased the expression levels of *IL6*, *IL8*, and *CCL2* genes, three classical markers of the proinflammatory M1 phenotype, compared to THP-1 cells exposed to the control conditioned media (Figure 6b–d).

The CMSC-conditioned media was also assessed for its capacity to support human umbilical vein endothelial cell (HUVEC) angiogenesis in vitro. The results show a significant reduction in the functional response of HUVECs as measured by several parameters (number of meshes, master segments and junctions, and total mesh area) when endothelial cells were cultured with the media conditioned by CMSCs from irradiated rats compared to the control (Figure 6e–i).

## 3. Discussion

Late-onset RIHDs can significantly affect the quality of life and increase the long-term mortality of cancer survivors who undergo thoracic radiotherapy, such as those who recover from left-sided breast cancer. Cardiac irradiation due to RT can cause concentric remodeling and restrictive cardiomyopathy, with predominant diastolic dysfunction [29,30,31], GLS reduction, and stiffening of the myocardium due to microvascular inflammation, cardiomyocyte hypertrophy, and interstitial deposition of collagen and fibrotic tissue [32]. The mechanisms underlying cardiac toxicity mediated by RT are complex and involve crosstalk between different cardiac cells, first those more sensitive to oxidative/inflammatory damage. Other known mechanisms involved in RT-induced cardiotoxicity are linked to vascular injury [24] and thrombotic events [12], with further consequent myocardial low-grade inflammation and interstitial fibrosis. To what extent interstitial fibrosis is a consequence of parenchymal cell death and inflammation compared to direct activation of the stromal compartment is not known yet.

The possible direct and early effects of cardiac irradiation during thoracic RT on phenotypic shifts in the resident population of CMSCs (excluding the immune compartment) have not been investigated yet. In fact, CMSCs are important players in cardiac homeostasis and tissue repair, with many subpopulations identified by specific markers and different functional states described thus far [16,33]. The subsets of CMSCs, as well as the expression of specific genes and markers, change significantly under stress (e.g., metabolic stress) and post-injury (e.g., post-myocardial infarction) [22,33,34]. Novel insights into the specific effects of heart irradiation during RT on the features and phenotype of CMSCs may help in understanding the triggers of cardiac remodeling and fibrosis after RT and the later development of LV dysfunction. Moreover, the understanding of early mechanisms of damage prior to clinically detectable effects or acute vascular events would be helpful in the development of possible adjuvant treatments that may counteract these mechanisms, with the final aim of reducing the overall burden of RIHDs [35].

Here, we have described how resident mesenchymal stromal cells isolated from a rat model of RT-induced cardiac dysfunction display an early change in the phenotypic and functional profiles, independent of any detectable functional alteration of the organ. In fact, this rat model develops dose-dependent cardiac dysfunction starting 12 months after the end of RT, when a significant reduction of GLS and microvascular density in the cardiac apex can be detected [23]. These features significantly resemble the pathophysiological manifestations of RIHD, in particular concerning the development of concentric remodeling and diastolic dysfunction, leading to heart failure with preserved ejection fraction [3,31]. Overall, the altered features of CMSCs described here represent a very early detectable sign of cardiotoxicity directly attributable to heart irradiation in a clinically relevant rat model. Indeed, the isolation of CMSCs for direct analysis allowed us to unveil early changes in their phenotype, particularly at the paracrine level, which could not have been detectable in the whole myocardial tissue due to dilution and confounding effects within the other cell types.

Our results indicate a reduction in the pool of resident non-activated mesenchymal stromal cells. This shift is characterized by impairment of typical mesenchymal functions, such as migration and 3D growth, and transcriptional changes. Interestingly, cells derived from hearts irradiated with the highest dose show a significant modulation over time in the transcription of genes included in GO terms related to TGFβ1 signaling. However, no alterations in specific myofibroblast activation or fibrotic YAP-mediated pathways were detectable in our conditions. Nonetheless, consistent with the GO term analysis, altered paracrine profiles and functional effects were clearly measurable, particularly concerning the overall profile of secreted cytokines, as well as an increased ability of CMSCs to polarize macrophages toward pro-inflammatory features and to reduce support to endothelial cells for angiogenesis, consistent with the pathogenetic mechanisms discussed above. The extent of these alterations appears to be more significant with increasing radiation doses and time of follow-up.

As mentioned above, the transcriptional change is associated with significant alterations in the paracrine functions of stromal cells at the 12-week follow-up point, where the profile of secreted cytokines appears overall modified and capable of increasing the expression of pro-inflammatory cytokines in macrophages. This is in line with the tight connection between inflammatory and fibrotic pathways in the pathophysiology of tissue fibrosis and remodeling [24,36]. Consistently, CMSCs from the highest radiation dose groups released lower levels of the fibrosis repressor PAI-1 and of the angiogenic factor VEGF. Concerning the latter, the reduction in stromal VEGF release is also consistent with the impaired paracrine support for angiogenesis by endothelial cells described here and, possibly, with the long-term results of reduced microvasculature density 12 months after the end of RT previously described in this same rat model [23].

One limitation of our study concerns the absence of any oncologic disease in the rat model of RT used. On the other hand, the lack of any local or systemic effect due to cancer progression could grant a more reproducible system for studying RT-specific effects without unpredictable variables. Another limitation is represented by the lack of longer follow-ups available for this study due to constraints on financial and personnel resources imposed by the large multicenter European grant that supported this project. In conclusion, the data reported here represents a consistent biological substrate of the delayed cardiac dysfunction phenotype observed in the thoracic RT rat model, with the development of delayed diastolic dysfunction [23]. This feature of RT-induced cardiotoxicity is susceptible to vascular ischemic damage and wall stress, particularly at the apical level, leading to an abnormal contraction pattern in the setting of an apparently normal ejection fraction [37]. We speculate that the low-grade activation of cardiac stromal cells, with a reduction in paracrine angiogenetic support and increased proinflammatory signals, may represent the early biological background of the cardiac functional events documented in vivo with later onset, supporting future developments in diagnostic markers and therapeutic targets [38]. To the best of our knowledge, this is the first evidence of an early specific effect of RT cardiotoxicity on the cardiac stromal compartment, affecting sub-clinically the myocardial microenvironment and its intercellular signaling profile.

## 4. Materials and Methods

### 4.1. Irradiation Procedure

Rats were anesthetized intraperitoneally with midazolam (4.76 mg/kg BW), medetomidine (0.356 mg/kg BW), and fentanyl (0.012 mg/kg BW). Sufficient sedation was verified by the absence of a paw withdrawal reflex. Animals were submitted to a CT simulation (Somatom Sensation, Siemens, Erlangen, Germany) consisting of helical CT scans with a 2 mm slice thickness and volumetric image reconstruction with an axial slice width of 1 mm. For a reproducible position, anesthetized rats were placed in an acrylic phantom in a supine position with fiducial markers (small radiopaque spheres) over the skin markers, allowing us to correlate their CT images to external anatomical references. Target structures (heart) and organs at risk (lungs and vertebral bodies) were delineated on the planning CT scan to virtually optimize the radiation beam delivery. A 3D computerized treatment planning system (XiO, Elekta, Computerised Medical Systems Inc., Saint Louis, MO, USA), applying its superposition algorithm and heterogeneity correction, was used to calculate the radiation absorbed dose distribution throughout the rat body, generated by a 6 MV photon beam incident perpendicularly to the rat’s chest, with an equivalent square field size of 2.3 cm by 2.3 cm at 5 cm depth from the phantom’s surface. The treatment plans were calculated for the prescribed single doses of 0.04, 0.3, and 1.2 Gy. Finally, each anesthetized rat inside the phantom was set up at the linear accelerator (LINAC) by aligning their fiducial markers with the LINAC light field and correlating the room lasers with the beam’s isocenter. Rats were irradiated one at a time, according to the computerized treatment plan and the prescribed dose to the heart (0.04, 0.3, or 1.2 Gy) in an Elekta Synergy S LINAC operating at a maximum dose rate of 600 MU per min. For each dose group, the treatment was administered for 23 consecutive days (weekends excluded). Control rats were sham-irradiated (0.0 Gy) following the same procedure for the irradiated experimental groups. The detailed irradiation procedure has been published [23]. Five rats were randomly allocated to each experimental group. After the end of RT, rats were housed for 6 or 12 weeks before sacrifice and organ collection. All rats survived the procedures; all experiments were carried out following the Guide for the Care and Use of Laboratory Animals, with prior approval by the institutional Animal Welfare Body, licensed by DGAV, the Portuguese competent authority for animal protection (license number 0421/000/000/2018). For organ collection, animals were deeply anesthetized and bled from the inferior vena cava; hearts were subsequently perfused with PBS to wash out circulating cells and then excised.

### 4.2. Explant and Cell Culture

Resident CMSCs were isolated by primary explant cultures. Whole atria and ventricle samples were mechanically cut into 1–2 mm^3^ fragments, washed in PBS, partially digested in trypsin-EDTA 0.5% (Gibco, Thermo Fisher Scientific Inc., Waltham, MA, USA) for 3 min, and finally plated as primary explant cultures on fibronectin (FN)-coated dishes (Cell Guidance Systems, Cambridge, UK). Explants were cultured in standard incubators in complete explant medium (CEM)—IMDM (Gibco), 1% penicillin—streptomycin, 1% L-glutamine, 0.1 mM β-mercaptoethanol, and 0.1% Primocin (InvivoGen, San Diego, CA, USA)—supplemented with 20% FBS (Gibco, Thermo Fisher Scientific Inc., Waltham, MA, USA). To evaluate the outgrowth yield, a scoring system was set up: score 0 = no or few cells within a 200 µm radius; score 1 = confluent cell outgrowth beyond a 200 µm radius (Figure 1a). After 3 weeks, CMSCs were collected as outgrowth explant-derived cells by gentle trypsinization [39] and frozen. Once thawed, CMSCs were plated on FN-coated dishes and used after one week for all experiments.

### 4.3. Cell Proliferation Assay

An AlamarBlue metabolic assay (Invitrogen, Thermo Fisher Scientific Inc., Waltham, MA, USA) was used to study cell proliferation. For each experimental group, 3 × 10^3^ CMSCs per well were plated in quintuplicate in a 96-well plate and cultured for 24 h in CEM 5% FBS. For each timepoint, cells were incubated for 4 h with a 10% AlamarBlue solution before data acquisition. Absorbance was measured at 560 nm and 630 nm by a Readwell Touch Automatic ELISA Plate Analyzer (Robonik, Ambarnath, Maharashtra, India). The first measurement (24 h after cell seeding) was used as the baseline (day 0), and results are quantified as the fold increase versus baseline level.

### 4.4. Scratch Assay

To evaluate cell migration, a scratch assay was performed. A total of 10^5^ CMSCs per well were plated in 12-well plates coated with fibronectin (Corning, Somerville, MA, USA) in CEM 10% FBS. The scratch was performed after 24 h, and then the cells were washed with PBS and cultured with CEM 2% FBS for 20 h. To evaluate CMSC migration capacity, images were captured after 14 and 20 h with a Nikon Eclipse Ti fluorescence microscope equipped with a motorized stage and NIS-Elements AR 4.30.02 software (Nikon Corporation, Tokyo, Japan). Images were analyzed using ImageJ software (Windows 64-bit Java 8 version, NIH, USA; available at: https://imagej.net/ij/download.html, accessed on 22 February 2024) by an automatic macro for scratch area measurement, normalized to the initial area at T0.

### 4.5. Spheroid Culture

For the spheroid-forming assay, 5 × 10^4^ CMSCs per well were plated in 12-well plates coated with poly-D-lysine (Corning, Somerville, MA, USA) in CEM supplemented with 10% FBS. Random images were captured after 1 week of culture with a Nikon Eclipse Ti microscope equipped with NIS-Elements AR 4.30.02 software (Nikon Corporation, Tokyo, Japan). Images were analyzed using ImageJ software (NIH, USA), exploiting the plugins for particle count and area measurement.

### 4.6. Flow Cytometry Analysis

A total of 3 × 10^5^ CMSCs per line were used for staining and flow cytometry analysis. Samples were incubated with primary antibodies at room temperature for 15 min. For indirect staining, cells were washed in FACS media (PBS + FBS 2%) after the first incubation, centrifuged for 5 min, resuspended with the secondary antibody, and incubated at room temperature for 20 min. The antibodies used were: anti-DDR2 (sc-81707, Santa Cruz Biotechnology, Inc., Dallas, TX, USA) + goat anti-mouse Alexa Fluor 647 (Invitrogen); PerCP/Cy5.5-CD90 (202515, BioLegend, San Diego, CA, USA); APC/Cy7-CD45 (202216, BioLegend, San Diego, CA, USA); and APC-CD31 (FAB3628A, R&D systems, Minneapolis, MN, USA). Sample acquisition was performed on a FACSAria™ II flow cytometer equipped with the 6.1.1 Diva software (BD Biosciences, Franklin lakes, NJ, USA). Data analysis was performed using FlowJo software, version 10.6.1.

### 4.7. RNA Extraction 

Total RNA was extracted using the miRNEasy mini kit (Qiagen, Venlo, The Netherlands) according to the manufacturer’s instructions with on-column DNAse treatment. All samples were extracted in parallel to reduce inter-sample variability. CMSC samples (2 × 10^5^ cells) were lysed in Qiazol and stored at −80 °C until extraction. RNA was quantified using a NanoDrop One/OneC Microvolume UV—Vis Spectrophotometer (Thermo Fisher Scientific, Waltham, MA, USA).

### 4.8. Real-Time Polymerase Chain Reaction (RT—PCR)

For each experimental condition, RNA (500 ng) was reverse transcribed using the high-capacity cDNA reverse transcription kit with random hexamers (Applied Biosystems, Thermo Fisher Scientific, Waltham, MA, USA) according to the manufacturer’s protocol. Tubulin was selected as the housekeeping gene using the Normfinder algorithm as the most stable reference among five candidates across all conditions. Primer sequences are reported in Supplemental Table S5. PCR was performed on a 7900 Fast Real-time PCR system (Applied Biosystems, Thermo Fischer Scientific, Waltham, MA, USA). Gene expression levels are plotted as 2^−∆ct^, unless specified. Plots were generated using GraphPad Prism version 8.

### 4.9. RNAseq and Data Analysis

We performed RNA-seq analysis on CMSC RNA samples at both the 6- and 12-week follow-ups. RNA (500 ng) was used for 3′-mRNA sequencing (Quanteseq 3′ mRNA—Lexogen, Vienna, Austria). Read collection, alignment, counts, and differential gene expression analysis were performed in service. We excluded genes with zero counts in all samples. We then selected those genes with a *p*-value adjusted to <0.05 in each comparison between experimental groups. Starting from counts per million of data (CPM), a heatmap was generated using the R package pheatmap (GNU Project). Euclidean distance was calculated using hclust clustering methods implemented in R software, version 3.6.3. The gene list obtained was used for Gene Ontology (GO), Kyoto Encyclopedia of Genes and Genomes (KEGG), WikiPathway (WP), and Reactome (REAC) analyses performed on the Gprofiler online platform. Starting from GO enrichment data, a ggplot was generated using R software, version 3.6.3.

### 4.10. Conditioned Media Analysis

Conditioned media from CMSC cultures were collected after 24 h of conditioning in CEM 0.1% FBS and stored at −80 °C until analysis. Secretome screening was performed by the Proteome Profiler Rat XL Cytokine Array (R&D System, Minneapolis, MN, USA) following the manufacturer’s instructions. Optical density analysis was performed by ImageLab Software version 6 (BioRad, Hercules, CA, USA).

### 4.11. Macrophage Polarization Assay

To evaluate the M1 polarization of macrophages, 2 × 10^5^ human monocytic THP-1 cells were plated in 24-well plates in DMEM 4.5 g/L glucose supplemented with 10% FBS, 1% penicillin—streptomycin, 1% L-glutamine, and 0.1 mM β-mercaptoethanol. THP-1 monocytes were differentiated into macrophages by 24 h of incubation with 50 nM phorbol 12-myristate 13-acetate (PMA), followed by 24 h of incubation in CMSC-conditioned media for each condition. As a positive control, macrophages were polarized toward M1 by incubation with 20 ng/mL IFN-γ (Peprotech, Thermo Fisher Scientific Inc., Waltham, MA, USA) and 1 µg/mL LPS (L2880, Sigma-Aldrich, Burlington, MA, USA). Total macrophage RNA was extracted and reverse transcribed to perform RT—PCR. The relative expression was calculated using the 2^−ΔΔCT^ method, using SHAM conditions as the reference, for the following genes: *CCL2*, *IL6*, and *IL8*. *GAPDH* was used as a housekeeping gene. Primer sequences are reported in Supplemental Table S1.

### 4.12. Angiogenesis Assay

Human umbilical vein endothelial cells (HUVECs) were plated (2.5 × 10^4^ cells/well) on Matrigel-coated 96-well plates (Growth Factor Reduced Matrigel Matrix Phenol Red Free, BD Biosciences, Franklin lakes, NJ, USA) and cultured for 18 h in the CMSC-conditioned media previously collected from all groups. Endothelial growth media (EGM, Lonza, Basel, Switzerland) was used as a positive experimental control. The Angiogenesis Analyzer Plugin of ImageJ Software (NIH) was used on randomly captured images with a 4× objective on a Nikon Eclipse TI inverted microscope (Nikon Corporation, Tokyo, Japan).

### 4.13. Soluble Collagen Assay

Soluble collagen was quantified using the Sircol Soluble Collagen Assay (S1000, Biocolor, Ltd., Carrickfergus, UK), according to the manufacturer’s instructions. The absorbance at 590 nm was recorded using the Varioskan™ LUX Multimode Reader (Thermo Fisher Scientific). The data were analyzed using the SkanIt software version 7.0 (Thermo Fisher Scientific Inc., Waltham, MA, USA).

### 4.14. Tunable Stiffness Substrate Preparation

PDMS substrates with tunable stiffness were produced using pure Sylgard 184 and Sylgard 527 (Dow Corning, Midland, MI, USA) mixed in different ratios. For 1 MPa, a solution of pure Sy184 was used, while for 100 kPa and 20 kPa, 1:10 and 1:30 Sy184:Sy527 were employed. Curing of approximately 3 mm thick films was performed by heating at 65 °C. Indentation of the specimens, immersed in demineralized water at room temperature (RT) or at 37 °C, was performed in displacement control (indentation depth = 2 µm, cantilever stiffness = 4.4 N/m for Sylgard 184, 0.5 N/m for the other specimens) using the PIUMA nanoindenter (Optics11 life, Amsterdam, The Netherlands), which automatically provided the load-indentation curves and their respective elastic modulus (E) values (Hertz model fitting). Each PDMS specimen was indented in three different regions (5 × 5 points, step size = 50 μm). Three different indentation velocities (v1 = 1 μm/s, v2 = 10 μm/s, v3 = 50 μm/s) were set to investigate the viscoelastic response of the specimens.

### 4.15. Statistical Analysis

Statistical analysis was performed by GraphPad Prism 8 software (GraphPad Software, San Diego, CA, USA). All results are presented as the mean value ± standard error of the mean (SEM). Parametric and nonparametric (as appropriate) one-way ANOVA followed by Bonferroni correction or Fisher’s LSD for multiple comparisons was used to test for statistical significance. *p* values <0.05 were considered significant.

## Data Availability

The datasets used and/or analyzed during the current study are available from the corresponding author on reasonable request.

## Supplementary Materials

The following supporting information can be downloaded at: https://www.mdpi.com/article/10.3390/ijms25052873/s1.

## Abbreviations

RT	radiotherapy
RIHD	radiation-induced heart disease
GLS	global longitudinal strain
CMSCs	cardiac mesenchymal stromal cells
ECM	extracellular matrix
PDMS	poly-dimethylsiloxane
HUVEC	human umbilical vein endothelial cell
