# Supplementary material for: Early Impairment of Paracrine and Phenotypic Features in Resident Cardiac Mesenchymal Stromal Cells after Thoracic Radiotherapy
Source: Int J Mol Sci. 2024 Mar 1;25(5):2873. doi: 10.3390/ijms25052873 (PMC10932029; doi:10.3390/ijms25052873)

Supplemental Material

Article

Early impairment of paracrine and phenotypic features in resident cardiac mesenchymal stromal cells after thoracic radiotherapy

Vittorio Picchio [1†], Roberto Gaetani [2†], Francesca Pagano [3], Yuriy Derevyanchuk [2], Olivia Pagliarosi [2], Erica Floris [4], Claudia Cozzolino [4], Giacomo Bernava [5], Antonella Bordin [4], Filipe Rocha [6], Ana RS Pereira [6], Augusto Ministro [6], Ana T Pinto [6], Elena De Falco [4,7], Gianpaolo Serino [8], Diana Massai [8], Radia Tamarat [9], Maurizio Pesce [5], Susana Constantino Rosa Santos[6], Elisa Messina, [2§*], Isotta Chimenti [4,7 §,*]

[1] Department of Angio Cardio Neurology, IRCCS Neuromed, Pozzilli, Italy.
[2] Department of Molecular Medicine, Sapienza University, Rome, Italy.
[3] Institute of Biochemistry and Cell Biology, National Council of Research (IBBC-CNR), Monterotondo (RM), Italy.
[4] Department of Medical Surgical Sciences and Biotechnologies, Sapienza University, Latina, Italy.
[5] Centro Cardiologico Monzino, IRCCS, Milano, Italy.
[6] Centro Cardiovascular da Universidade de Lisboa (CCUL@RISE), Lisbon School of Medicine of the Universidade de Lisboa; Lisbon, Portugal.
[7] Mediterranea Cardiocentro, Napoli, Italy.
[8] Department of Mechanical and Aerospace Engineering, Politecnico di Torino, Turin, Italy; Interuniversity Center for the Promotion of the 3Rs Principles in Teaching and Research, Italy.
[9] Institut de Radioprotection et de Sûreté Nucléaire (IRSN), Fontenay-aux-Roses, France.
* Correspondence: Phone: +3907731757234. E-mail: elisa.messina@uniroma1.it; isotta.chimenti@uniroma1.it
† Equal contribution.
§ Equal contribution.

Content of this appendix:

Supplemental Tables **page 2**

Supplemental Figures **page 10**

Supplemental Table S1. Flow cytometry data (Mean +/- SEM) plotted in figure 3.

	**6 Weeks**	
	**CD90+/DDR2-**	**CD31+/CD45-**
**SHAM**	39,1 +/- 5,1	2,2 +/- 0,4
**0.04 Gy**	46,9 +/- 7,7	2,0 +/- 0,4
**0.3 Gy**	35,5 +/- 11,2	1,6 +/- 0,4
**1.2 Gy**	21,4 +/- 5,9	1,2 +/- 0,3
	**12 Weeks**	
	**CD90+/DDR2-**	**CD31+/CD45-**
**SHAM**	49,4 +/- 7,5	0,6 +/- 0,2
**0.04 Gy**	45,4 +/- 7,7	0,4 +/- 0,0
**0.3 Gy**	49,6 +/- 6,3	0,5 +/- 0,0
**1.2 Gy**	52,6 +/- 12,9	0,2 +/- 0,0

Supplemental Table S2. Gene Ontology (GO) with statistical analysis of modulated genes from RNA-seq data. GO-terms that are statistically significant from the gene ontology analysis on the gProfiler database, on the comparison between cells from the sham and 1.2 Gy at the 12-week follow-up.

**GO Molecular Function**	**Description**	**Count in network**	**Adjusted P-value**	**Intersection**
GO:0005198	structural molecule activity	5 of 801	$1,2x10^{-2}$	COL11A1, AC105662.1, RPS3A, RPL19, RPS23
GO:0003735	structural constituent of ribosome	2 of 4	$3,04\times10^{-2}$	AC105662.1, RPS3A, RPL19, RPS23
**GO Cellular Component**	**Description**	**Count in network**	**Adjusted P-value**	**Intersection**
GO:0042788	polysomal ribosome	2 of 23	$7,743x10^{-3}$	RPL19, RPS23
GO:0005829	cytosol	8 of 3141	$1.924x10^{-2}$	S100B, RBP1, AC105662.1, RPL19, GDA, STMN1, RPS23, SRGAP2
GO:0005737	cytoplasm	13 of 8997	2.205×10^{-2}	CREB3L1, S100B, RBP1, PFN1, AC105662.1, SLC25A5, RPL19, GDA, STMN1, RPS23, SRGAP2, FNBP1, SQOR
GO:0005840	ribosome	4 of 591	3.148×10^{-2}	AC105662.1, RPS3A, RPL19, RPS23
GO:0005844	polysome	2 of 57	4.828×10^{-2}	RPL19, RPS23
**KEGG**	**Description**	**Count in network**	**Adjusted P-value**	**Intersection**
KEGG:03010	Ribosome	3 of 158	4.838×10^{-3}	RPS3A, RPL19, RPS23
**REACTOME**	**Description**	**Count in network**	**Adjusted P-value**	**Intersection**
R-RNO-927802	Nonsense-Mediated Decay (NMD)	3 of 141	9.308×10^{-3}	RPS3A, RPL19, RPS23
R-RNO-72312	rRNA processing	3 of 201	$2.655x10^{-2}$	RPS3A, RPL19, RPS23
R-RNO-72766	Translation	3 of 244	4.688×10^{-2}	RPS3A, RPL19, RPS23
**WIKIPATHWAY**	**Description**	**Count in network**	**Adjusted P-value**	**Intersection**
WP30	Cytoplasmic Ribosomal Proteins	3 of 80	1.882×10^{-4}	RPS3A, RPL19, RPS23
WP590	Cardiovascular Signaling	2 of 36	2838×10^{-3}	COL11A1, STMN1

Supplementary Table S3. Gene Ontology (GO) with statistical analysis of modulated genes from RNA-seq data. Selected GO-terms from the gProfiler database that are statistically significant with time (6 versus 12 weeks of follow-up) in the comparison between cells of the 1.2 Gy dose groups.

**GO-ter**<br><br>**Biological Process**	**Description**	**Count in network**	**Adjusted P-value**	**Intersection**
GO:0007179	TGF-β receptor signaling pathway	5 of 156	1.837×10-3	ZMIZ1, CCL2, LOX, VASN, DAB2
GO:0071560	Cellular response to TGF-β stimulus	5 of 206	3,578x10-3	ZMIZ1, CCL2, LOX, VASN, DAB2
GO:0071559	Response to TGF-β	5 of 209	3,655x10-3	ZMIZ1, CCL2, LOX, VASN, DAB2
GO:0071363	Cellular response to growth factor stimulus	7 of 562	5,003x10-3	ZMIZ1, CCL2, PDE8A, LOX, BCAR1, VASN, DAB2
GO:0071363	Enzyme linked receptor protein signaling pathway	8 of 798	4,362x10-3	ZMIZ1, CCL2, CDK4, LOX, BCAR1, VASN, ESM1, DAB2
GO:0070848	Response to growth factor	7 of 577	6,337x10-3	ZMIZ1, CCL2, PDE8A, LOX, BCAR1, VASN, DAB2
**GO Term**<br><br>**Cellular Component**	**Description**	**Count in network**	**Adjusted P-value**	**Intersection**
GO:0005737	Cytoplasm	28 of 8997	9,348x10-6	TUBA1A, SQOR, LIPA, MICAL3, RPL19, ZMIZ1, SERPINI1, CCL2, CDK4, FH, LOC108348287, ADM, FABP3, BCAR1, TOMM6, VASN, MCU, AABR07040892.1, DNAJC19, FNBP1, LIG4, LOC100363268, PPP2R3C, S100A4, SRGAP2, TSNAX, EMC2, DAB2
GO:0043229	Intracellular organelle	30 of 10611	2,486x10-4	TUBA1A, SQOR, LIPA, MGLL, MICAL3, RPL19, ZMIZ1, SERPINI1, CCL2, CDK4, FH, LOC108348287, RGD1561590, BCAR1, TOMM6, VASN, MCM4, MCU, AABR07040892.1, DIDO1, DNAJC19, FNBP1, LIG4, LOC100363268, PPP2R3C, S100A4, SRGAP2, TSNAX, EMC2, DAB2
GO:0043231	Intracellular membrane-bounded organelle	26 of 9156	1,037x10-3	TUBA1A, SQOR, LIPA, MGLL, MICAL3, RPL19, ZMIZ1, SERPINI1, CCL2, CDK4, FH, RGD1561590, TOMM6, VASN, MCM4, MCU, DIDO1, DNAJC19, LIG4, LOC100363268, PPP2R3C, S100A4, SRGAP2, TSNAX, EMC2, DAB2
**KEGG**	**Description**	**Count in network**	**Adjusted P-value**	**Intersection**
KEGG: 04218	Cellular senescence	3 of 170	4,254x10-2	RT1-A1, CDK4, MCU
**WIKIPATHWAY**	**Description**	**Count in network**	**Adjusted P-value**	**Intersection**
WP: WP348	G1 to S cell cycle control	2 of 65	4,648x10-2	CDK4, MCM4

Supplemental Table S4. Gene Ontology analysis of the top 15 up-regulated and top 15 down-regulated cytokines obtained from the STRING database, with strenght >1, and involving at least 5 counts in the network.

term ID Biological Process	term description	observed count	strength	FDR	matching proteins in the network
GO:0033993	Response to lipid	18	1,04	5.46e-12	PAI-1, Adiponectin, CXCL7, Cystatin C, CCL2, IGFBP-3, IL-6, IL-17A, Lipocalin-2, CD106, RBP4, CCL20, MMP-2, IGFBP-2, MMP-9, CD54, GM-CSF, Osteopontin
GO:0034097	Response to cytokine	15	1,05	6.16e-10	PAI-1, Adiponectin, CXCL7, CCL2, IL-6, IL-17A, Lipocalin-2, CD106, CCL20, MMP-2, MMP-9, CD54, GM-CSF, CD10, Osteopontin
GO:0071345	Cellular response to cytokine stimulus	14	1,08	1.21e-09	PAI-1, CXCL7, CCL2, IL-6, IL-17A, Lipocalin-2, CD106, CCL20, MMP-2, MMP-9, CD54, GM-CSF, CD10, Osteopontin
GO:0030334	Regulation of cell migration	14	1,02	6.42e-09	Resistin, PAI-1, Adiponectin, FBLN3, CCL2, WISP-1, IGFBP-3, CCN3, MMP-3, CCL20, MMP-2, MMP-9, NT-3, CD54
GO:0071396	Cellular response to lipid	13	1,16	1.09e-09	PAI-1, CXCL7, CCL2, IGFBP-3, IL-6, IL-17A, Lipocalin-2, CCL20, MMP-2, MMP-9, CD54, GM-CSF, Osteopontin
GO:1901652	Response to peptide	12	1,15	7.63e-09	Resistin, PAI-1, Adiponectin, CCL2, IGFBP-3, IL-6, CD106, RBP4, MMP-2, MMP-9, CD54, AHSG
GO:0031667	Response to nutrient levels	12	1,1	2.22e-08	PAI-1, Adiponectin, Cystatin C, CCL2, IGFBP-3, IL-6, Lipocalin-2, CD106, IGFBP-2, MMP-9, CD54, Osteopontin
GO:0009617	Response to bacterium	12	1,01	1.99e-07	PAI-1, Adiponectin, CXCL7, CCL2, IGFBP-3, IL-6, Lipocalin-2, CD106, CCL20, MMP-9, CD54, GM-CSF
GO:0032496	Response to lipopolysaccharide	11	1,27	4.80e-09	PAI-1, CXCL7, CCL2, IGFBP-3, IL-6, Lipocalin-2, CD106, CCL20, MMP-9, CD54, GM-CSF
GO:0007568	Aging	11	1,24	7.63e-09	PAI-1, CCL2, IGFBP-3, IL-6, CD106, Clusterin, MMP-2, IGFBP-2, MMP-9, CD54, CD10
GO:0042493	Response to drug	11	1,12	9.35e-08	PAI-1, Adiponectin, Cystatin C, CCL2, IGFBP-3, IL-6, Lipocalin-2, MMP-2, IGFBP-2, MMP-9, CD54
GO:0010035	Response to inorganic substance	11	1,02	6.54e-07	PAI-1, Cystatin C, IL-6, Lipocalin-2, CD106, Clusterin, MMP-2, IGFBP-2, MMP-9, CD54, GM-CSF
GO:0006954	Inflammatory response	10	1,11	5.63e-07	CXCL7, CCL2, IL-6, IL-17A, CD106, CCL20, Clusterin, CD54, AHSG, Osteopontin
GO:0030335	Positive regulation of cell migration	10	1,09	8.28e-07	Resistin, PAI-1, CCL2, WISP-1, MMP-3, CCL20, MMP-2, MMP-9, NT-3, CD54
GO:0071347	Cellular response to interleukin-1	9	1,71	1.79e-10	PAI-1, CCL2, IL-6, IL-17A, Lipocalin-2, CCL20, MMP-2, MMP-9, CD54
GO:0071222	Cellular response to lipopolysaccharide	9	1,45	1.10e-08	PAI-1, CXCL7, CCL2, IL-6, Lipocalin-2, CCL20, MMP-9, CD54, GM-CSF
GO:0032868	Response to insulin	9	1,36	4.95e-08	Resistin, PAI-1, Adiponectin, CCL2, IGFBP-3, IL-6, RBP4, CD54, AHSG
GO:0001666	Response to hypoxia	9	1,21	5.78e-07	PAI-1, Adiponectin, Cystatin C, CCL2, IGFBP-3, CD106, MMP-2, MMP-9, CD54
GO:0048608	Reproductive structure development	9	1,06	7.46e-06	PAI-1, Cystatin C, CD106, RBP4, MMP-2, CD54, GM-CSF, CD10, AHSG
GO:0034612	Response to tumor necrosis factor	8	1,41	1.88e-07	Adiponectin, CCL2, IL-6, Lipocalin-2, CD106, CCL20, MMP-9, CD54

GO:0042593	Glucose homeostasis	8	1,34	5.25e-07	PAI-1, Adiponectin, CCL2, WISP-1, IL-6, CD106, RBP4, CD54
GO:0048545	Response to steroid hormone	8	1,09	2.07e-05	PAI-1, Adiponectin, CCL2, IL-6, IL-17A, IGFBP-2, CD54, Osteopontin
GO:0001819	Positive regulation of cytokine production	8	1,09	2.17e-05	PAI-1, Adiponectin, CCL2, IL-6, IL-17A, CCL20, Clusterin, GM-CSF
GO:0009314	Response to radiation	8	1,03	4.94e-05	CCL2, CD106, RBP4, Clusterin, MMP-2, MMP-9, CD54, CD10
GO:0048660	Regulation of smooth muscle cell proliferation	7	1,45	9.94e-07	Resistin, Adiponectin, WISP-1, IGFBP-3, IL-6, MMP-2, MMP-9
GO:0008284	Positive regulation of cell population proliferation	15	1	2.28e-09	Resistin, PAI-1, FBLN3, Cystatin C, CCL2, WISP-1, IL-6, CD106, Clusterin, MMP-2, IGFBP-2, MMP-9, NT-3, GM-CSF, FLT3LG
GO:0071356	Cellular response to tumor necrosis factor	7	1,41	1.60e-06	CCL2, IL-6, Lipocalin-2, CD106, CCL20, MMP-9, CD54
GO:0032355	Response to estradiol	7	1,33	4.29e-06	Cystatin C, CCL2, IGFBP-3, IL-6, MMP-2, IGFBP-2, MMP-9
GO:0050900	Leukocyte migration	7	1,31	5.74e-06	CXCL7, CCL2, IL-17A, CD106, CCL20, CD54, Osteopontin
GO:0007565	Female pregnancy	7	1,28	9.11e-06	PAI-1, Cystatin C, CCL2, IGFBP-3, MMP-2, IGFBP-2, MMP-9
GO:0051384	Response to glucocorticoid	7	1,27	1.08e-05	PAI-1, Adiponectin, CCL2, IL-6, IL-17A, IGFBP-2, CD54
GO:0046883	Regulation of hormone secretion	7	1,2	2.63e-05	Resistin, Adiponectin, IGFBP-3, CCN3, IL-6, RBP4, Osteopontin
GO:0050727	Regulation of inflammatory response	7	1,15	5.11e-05	PAI-1, Adiponectin, WISP-1, CCN3, IL-6, IL-17A, AHSG
GO:1901653	Cellular response to peptide	7	1,12	7.12e-05	PAI-1, Adiponectin, CCL2, IL-6, CD106, CD54, AHSG
GO:0097305	Response to alcohol	7	1,1	9.03e-05	PAI-1, Adiponectin, CCL2, CD106, RBP4, MMP-9, CD54
GO:0032103	Positive regulation of response to external stimulus	7	1,07	0.00014	PAI-1, CCL2, WISP-1, IL-6, IL-17A, MMP-2, NT-3
GO:0006979	Response to oxidative stress	7	1,03	0.00024	PAI-1, Adiponectin, Cystatin C, IL-6, Lipocalin-2, MMP-2, MMP-9
GO:0034614	Cellular response to reactive oxygen species	6	1,41	1.54e-05	PAI-1, Cystatin C, IL-6, Lipocalin-2, MMP-2, MMP-9
GO:0043200	Response to amino acid	6	1,38	2.01e-05	CCL2, MMP-3, IL-6, IL-17A, MMP-2, CD54
GO:0060326	Cell chemotaxis	6	1,33	3.50e-05	CXCL7, CCL2, CCN3, CD106, CCL20, Osteopontin
GO:0002685	Regulation of leukocyte migration	6	1,28	6.36e-05	PAI-1, CCL2, CCN3, CCL20, MMP-9, CD54
GO:0045471	Response to ethanol	6	1,24	9.30e-05	Adiponectin, CCL2, CD106, RBP4, MMP-9, CD54
GO:0009743	Response to carbohydrate	6	1,22	0.00012	PAI-1, Adiponectin, Cystatin C, CCL2, CD106, CD54
GO:0001503	Ossification	6	1,2	0.00015	WISP-1, IGFBP-3, MMP-2, MMP-9, AHSG, Osteopontin

GO:0007584	Response to nutrient	6	1,18	0.00019	Adiponectin, CCL2, CD106, IGFBP-2, MMP-9, Osteopontin
GO:0050730	Regulation of peptidyl-tyrosine phosphorylation	6	1,18	0.00019	Adiponectin, IL-6, NT-3, CD54, GM-CSF, AHSG
GO:0009612	Response to mechanical stimulus	6	1,16	0.00024	PAI-1, CCL2, IL-6, MMP-2, IGFBP-2, MMP-9
GO:0008406	Gonad development	6	1,15	0.00027	PAI-1, Cystatin C, RBP4, MMP-2, CD54, AHSG
GO:0071496	Cellular response to external stimulus	6	1,06	0.00068	PAI-1, IL-6, Lipocalin-2, CD106, MMP-9, CD54
GO:0001655	Urogenital system development	6	1,05	0.00079	Adiponectin, RBP4, MMP-2, MMP-9, CD10, Osteopontin
GO:0010712	Regulation of collagen metabolic process	5	1,82	2.64e-06	Resistin, PAI-1, Cystatin C, CCL2, IL-6
GO:0014910	Regulation of smooth muscle cell migration	5	1,57	2.71e-05	Resistin, PAI-1, Adiponectin, WISP-1, IGFBP-3
GO:0097530	Granulocyte migration	5	1,56	2.94e-05	CXCL7, CCL2, IL-17A, CCL20, Osteopontin
GO:0071385	Cellular response to glucocorticoid stimulus	5	1,54	3.78e-05	PAI-1, CCL2, IL-6, IL-17A, CD54
GO:0048661	Positive regulation of smooth muscle cell proliferation	5	1,47	7.17e-05	Resistin, WISP-1, IL-6, MMP-2, MMP-9
GO:0032526	Response to retinoic acid	5	1,41	0.00012	CCL2, RBP4, MMP-2, IGFBP-2, MMP-9
GO:0002687	Positive regulation of leukocyte migration	5	1,37	0.00017	PAI-1, CCL2, CCL20, MMP-9, CD54
GO:1901655	Cellular response to ketone	5	1,35	0.00022	PAI-1, CCL2, IL-6, CD54, Osteopontin
GO:0042542	Response to hydrogen peroxide	5	1,33	0.00025	PAI-1, Cystatin C, IL-6, Lipocalin-2, MMP-2
GO:0009749	Response to glucose	5	1,25	0.00053	PAI-1, Adiponectin, CCL2, CD106, CD54
GO:0010038	Response to metal ion	7	1	0.00034	PAI-1, IL-6, CD106, Clusterin, IGFBP-2, MMP-9, CD54
GO:0050731	Positive regulation of peptidyl-tyrosine phosphorylation	5	1,22	0.00073	Adiponectin, IL-6, NT-3, CD54, GM-CSF
GO:1903039	Positive regulation of leukocyte cell-cell adhesion	5	1,2	0.00082	CCL2, IL-6, CD106, IGFBP-2, CD54
GO:0031099	Regeneration	5	1,13	0.0017	PAI-1, CCL2, CCN3, MMP-2, AHSG
GO:2001234	Negative regulation of apoptotic signaling pathway	5	1,13	0.0017	PAI-1, Clusterin, MMP-9, CD54, GM-CSF
GO:0031668	Cellular response to extracellular stimulus	5	1,11	0.0020	IL-6, Lipocalin-2, CD106, MMP-9, CD54
GO:0010951	Negative regulation of endopeptidase activity	5	1,09	0.0024	PAI-1, Cystatin C, IL-6, MMP-9, AHSG
GO:0031349	Positive regulation of defense response	5	1,09	0.0024	PAI-1, WISP-1, IL-6, IL-17A, MMP-2

GO:0030336	Negative regulation of cell migration	5	1,08	0.0026	PAI-1, Adiponectin, CCL2, IGFBP-3, CCN3
GO:0071375	Cellular response to peptide hormone stimulus	5	1,08	0.0026	PAI-1, Adiponectin, CCL2, IL-6, AHSG
GO:1902105	Regulation of leukocyte differentiation	5	1,05	0.0033	Adiponectin, IL-6, IL-17A, GM-CSF, FLT3LG
GO:0051251	Positive regulation of lymphocyte activation	5	1,05	0.0036	CCL2, IL-6, CD106, IGFBP-2, FLT3LG
GO:0070372	Regulation of erk1 and erk2 cascade	5	1,02	0.0043	Adiponectin, CCL2, IL-6, CCL20, CD54
GO:0072001	Renal system development	5	1,02	0.0043	Adiponectin, RBP4, MMP-9, CD10, Osteopontin
GO:0022407	Regulation of cell-cell adhesion	6	1	0.0014	Adiponectin, CCL2, IL-6, CD106, IGFBP-2, CD54

Supplemental Table S5. Sequence of all primers used for real-time PCR.

**Rat Primer name**	**Sequence**
***COL1A1* Forward**	CTGACGCATGGCCAAGAAGA
***COL1A1* Reverse**	CCGTGCCATTGTGGCAGATA
***GATA4* Forward**	ATCTCACTATGGGCACAGCAG
***GATA4* Reverse**	ACTGGATGGATGGAGGACCC
***TGFB1* Forward**	TCCATGACATGAACCGACCC
***TGFB1* Reverse**	TGCCGTACACAGCAGTTCTT
***TUB1A1* Forward**	GCTGTAAGAAGCAACACCTCC
***TUB1A1* Reverse**	CCGACGTGGATGGAGATACA
***YAP* Forward**	AGGGCCTCTTCCTGATGGAT
***YAP* Reverse**	GTGATCCTCTGGTTCATGGCA
***ANKDR1* Forward**	CTTGGCGATCGTGGAGAAGT
***ANKDR1* Reverse**	CCAGTGGATGGCTGTGGATT
***ACTA2* Forward**	CATCACCAACTGGGACGACA
***ACTA2* Reverse**	TCCGTTAGCAAGGTCGGATG
***CTGF* Forward**	ACCCAACTATGATGCGAGCC
***CTGF* Reverse**	CATTGGTAACCCGGGTGGAG
***CYR61* Forward**	AGTTCCACCGCTCTGAAAGG
***CYR61* Reverse**	AAGCTCTCCCCGTTCTGGTA
**Human Primer name**	**Sequence**
***CCL2* Forward**	AGA GGC TGA GAC TAA CCC AGA
***CCL2* Reverse**	TTT CAT GCT GGA GGC GAG AG
***IL6* Forward**	AGT GAG GAA CAA GCC AGA GC
***IL6* Reverse**	ATT TGT GGT TGG GTC AGG GG
***IL8* Forward**	TGTACTCATGACCAGAAAGACC
***IL8* Reverse**	GGACACTACTGGGAGTGACAA
***GAPDH* Forward**	ATCAGCAATGCCTCCTGCAC
***GAPDH* Reverse**	TGGCATGGACTGTGGTCATG

Supplemental Figure S1. Quantification by flow cytometry of the CD45+ cell fraction in all samples, confirming their non-hematopoietic nature at the 6- **(a)** and 12-week **(b)** follow-up.

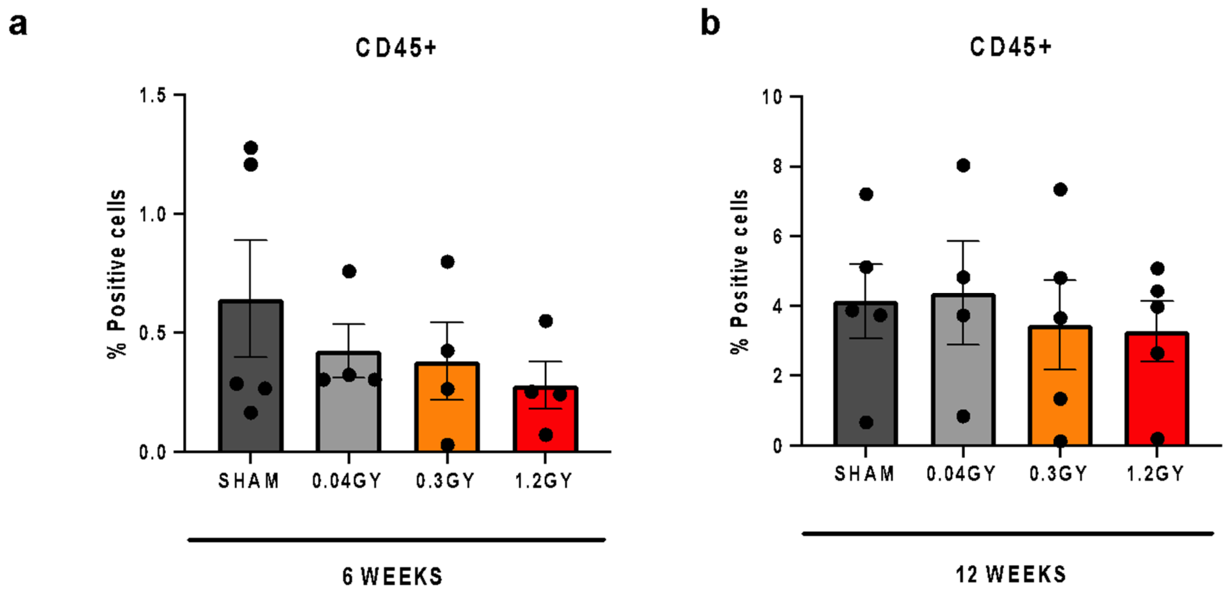

Supplemental Figure S2. Alamarblue cell proliferation assay was performed on cells isolated from all dose groups at both 6 **(A)** and 12-week follow-up **(B).** N=5. Quantification of the spheroid size in terms of average spheroid area calculation from random images, at the 6 **(C)** and 12-week follow-up **(D).** N>=4 per condition.

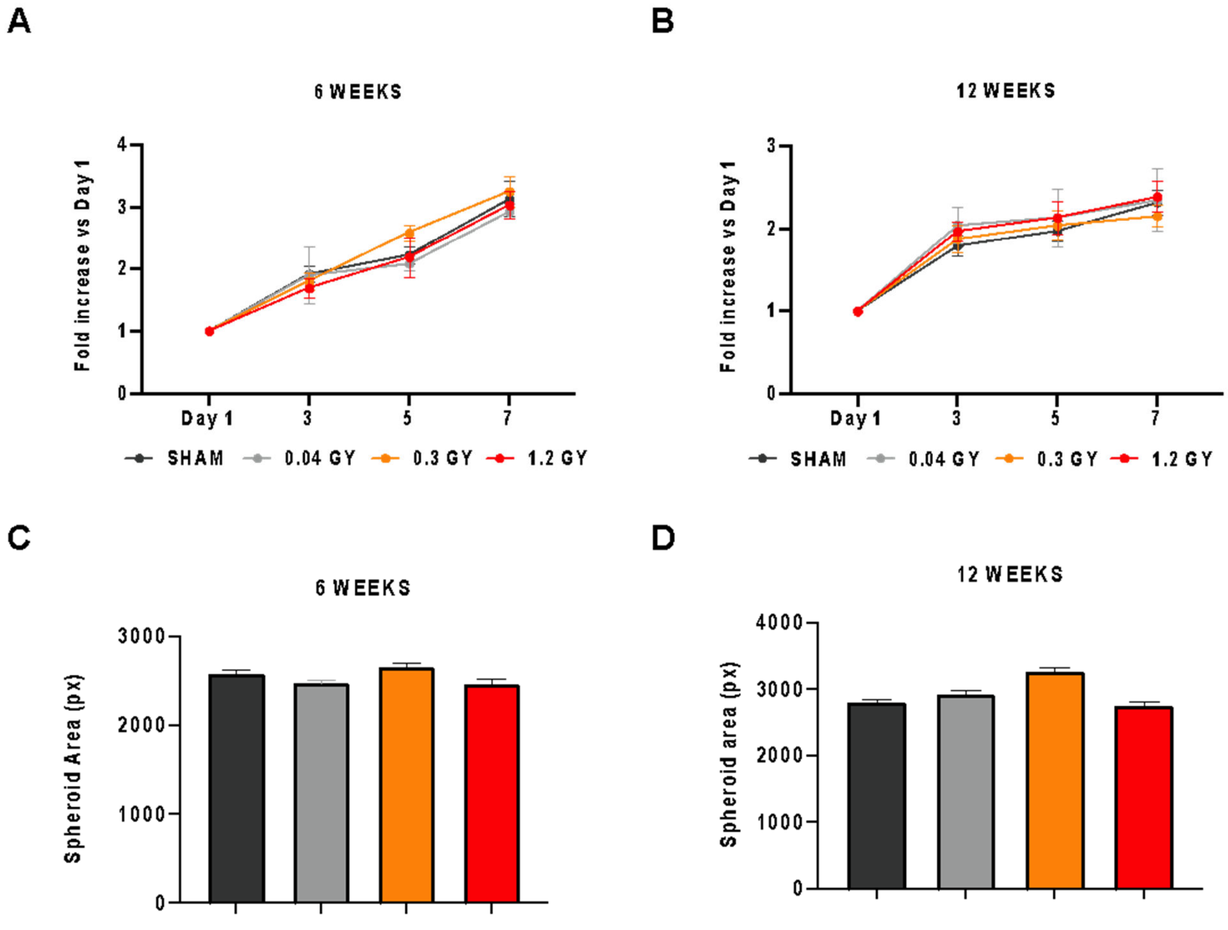

Supplemental Figure S3. Representative microscopy images, with corresponding binary masks created by the ImageJ analysis macro, of the migration assay 0 and 14 hours after creation of the scratch.

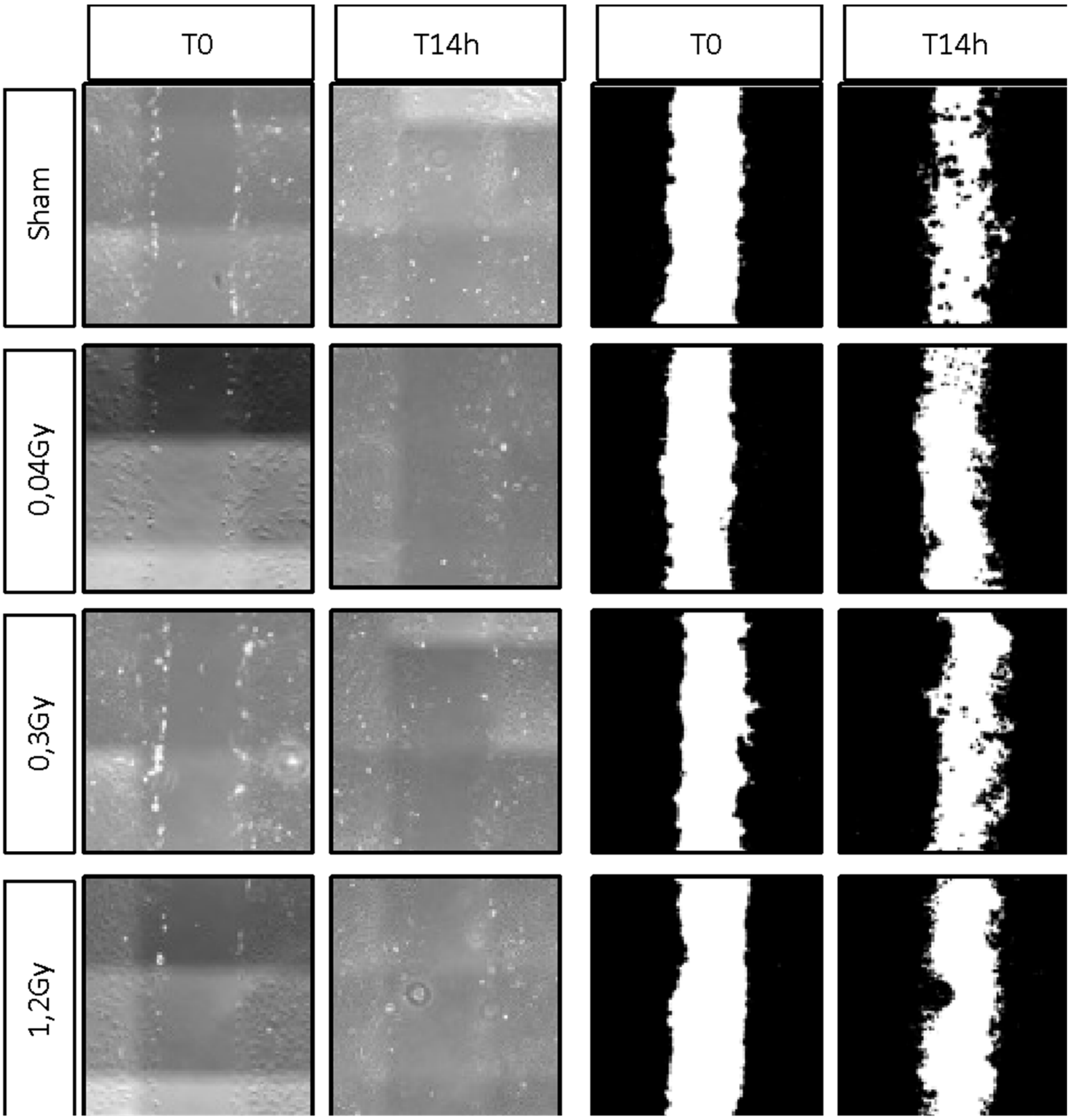

Supplemental Figure S4. Principal component analysis of transcriptomic data obtained from RNA-seq of whole ventricular tissue from all treatment groups at the two follow-up times of 6 and 12 weeks after the end of radiation.

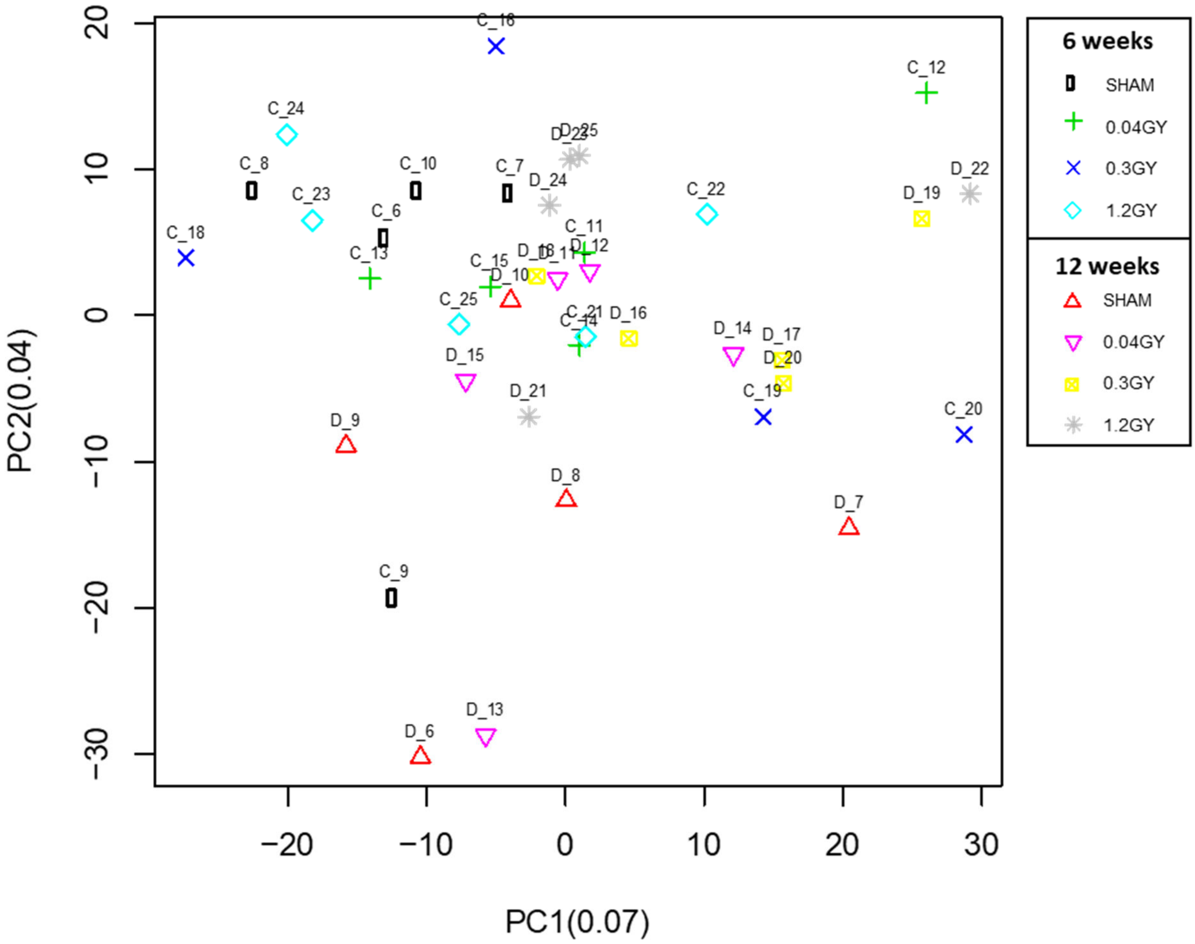

Supplemental Figure S5. Gene expression levels validated by qRT-PCR of selected RNA-seq hits from the transcriptomic data reported in figures 3 and 4. * p<0.05. ** p<0.01.

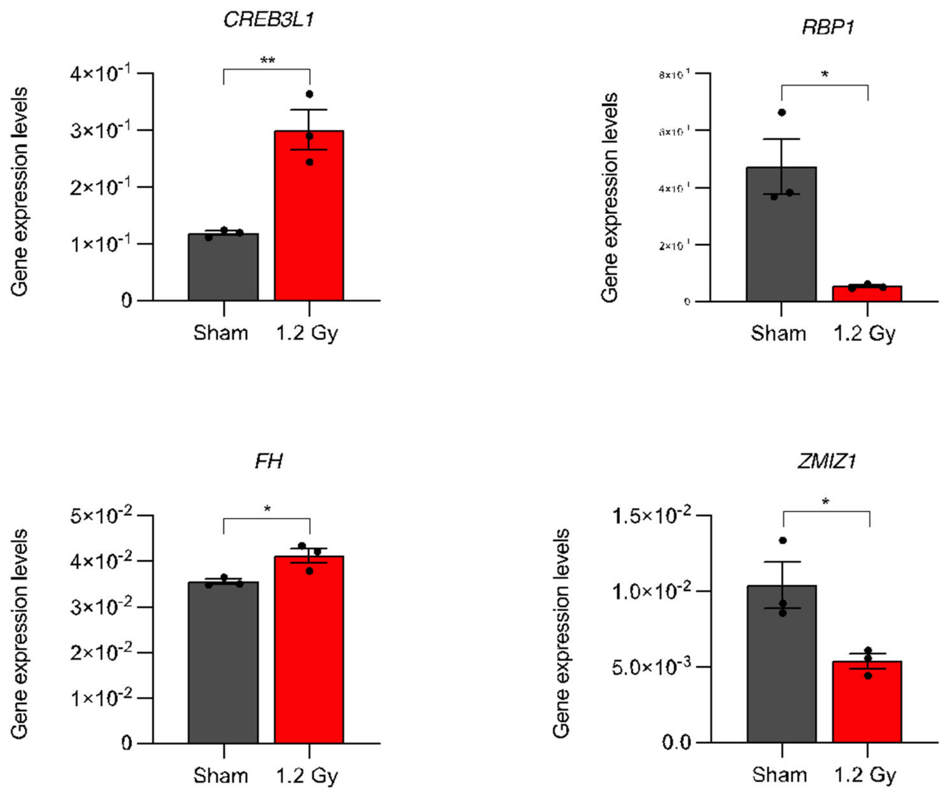

Supplemental Figure S6. Quantification of soluble collagen concentration by Sirius Red assay, released in conditioned media by CMSCs of the 12 follow-up after 24h (**a**) Quantification of the percentage of alpha smooth muscle actin (aSMA)-positive cells (**b**) from the 12-week follow-up groups, when cultured on substrates with different stiffness (1MPa, 100kPa, 20kPa). Representative immunofluorescence images (**c**) and corresponding quantification of the cytoplasm-to-nucleus YAP expression ratio (**d**) for CMSCs from the 12-week follow-up. Normalized gene expression levels (**e**) of the YAP-target genes *ACTA2*, *ANKDR1*, *CTGF*, *CYR61* in CMSCs from different irradiation groups (12-week follow-up), in relation to differential substrate stiffness values.

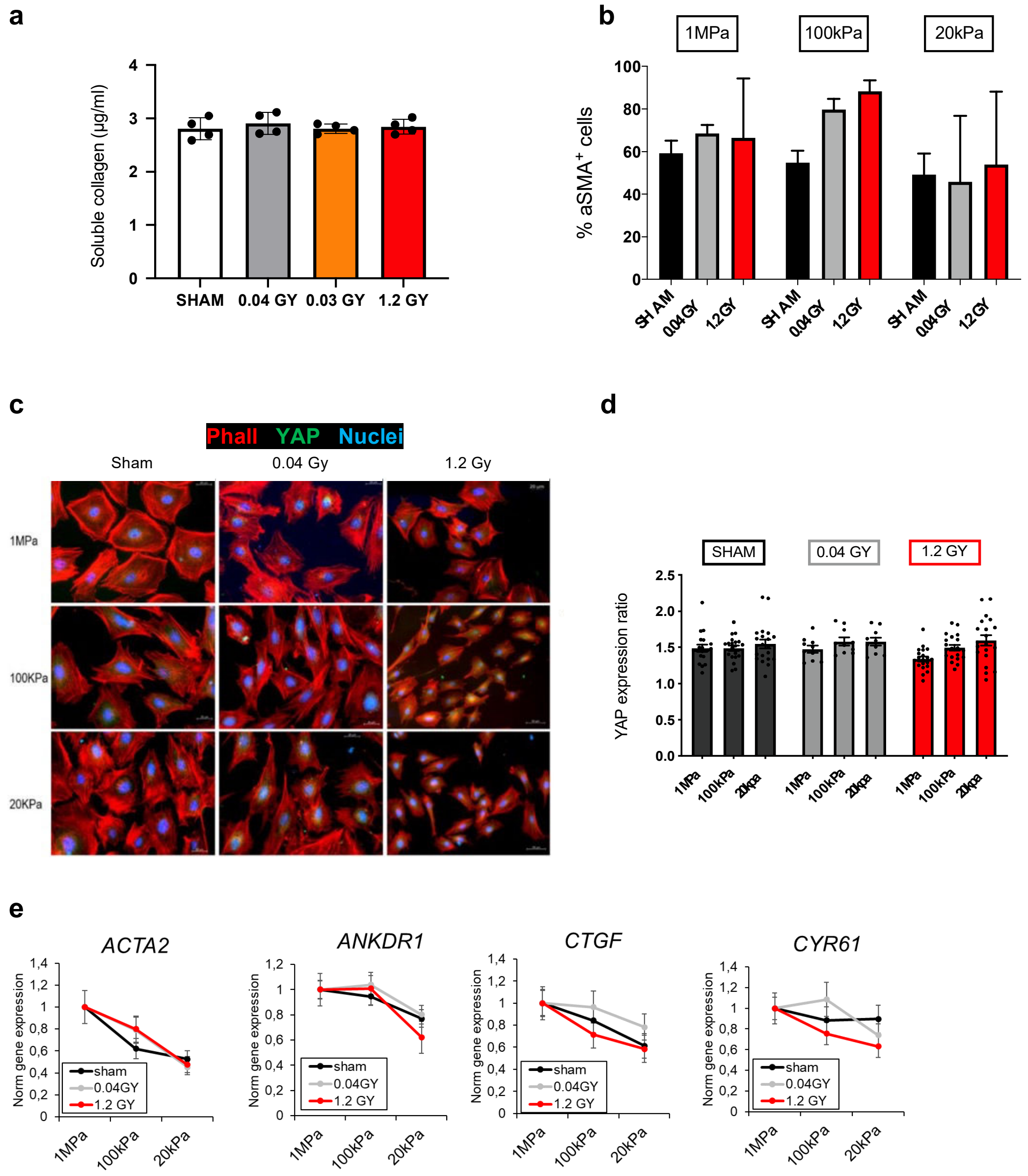

Supplemental Figure S7. STRING association networks of the highly modulated cytokines with the superimposition of a selection of GO terms of interest, related to cell migration, inflammation, aging, or radiation.

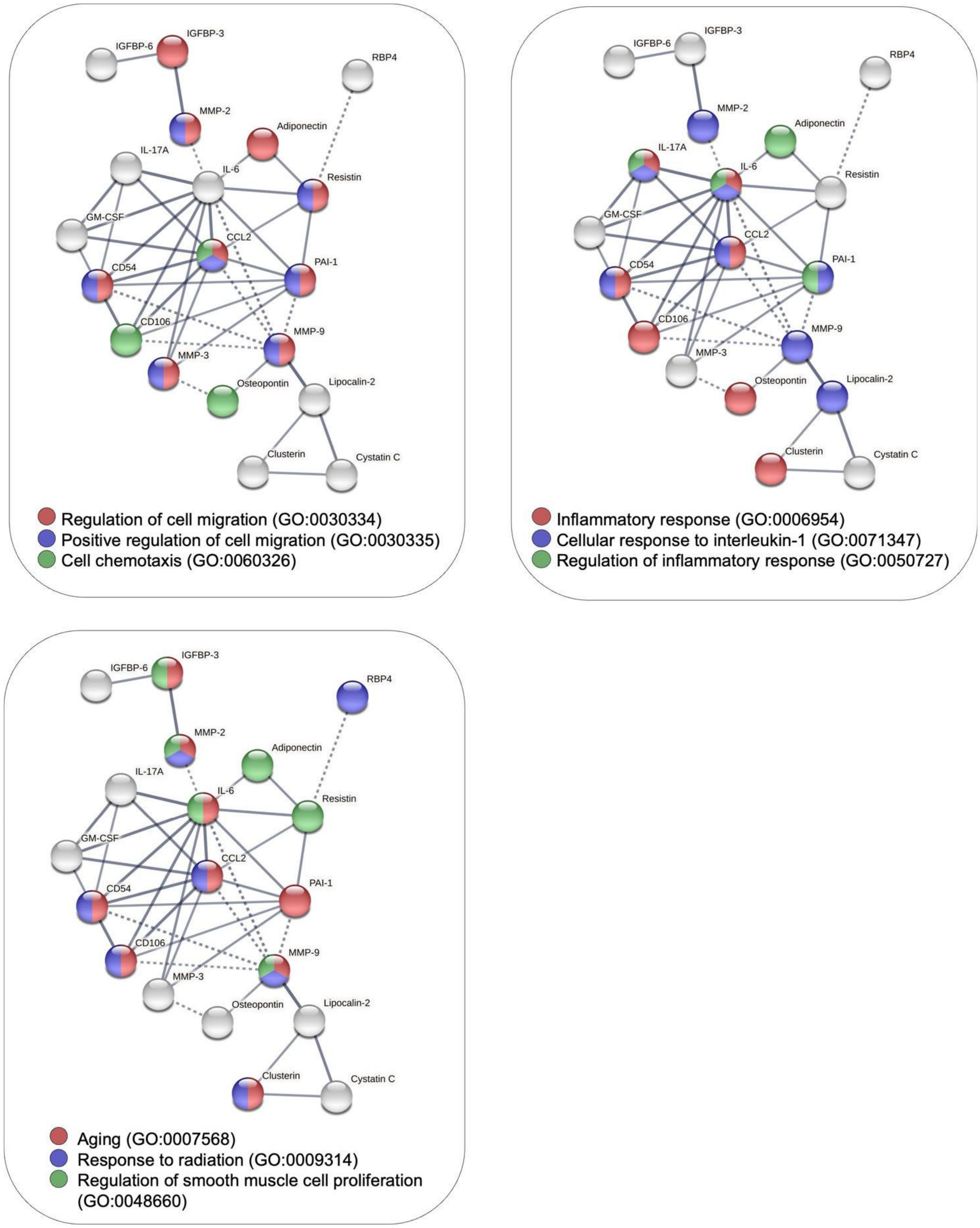

Supplement: Supplementary file 1 [file ijms-25-02873-s001.zip › ijms-2785718-supplementary.pdf]
